# The Association Between Hearing and Driving Performance Among Older Adults

**DOI:** 10.1093/geroni/igaf122.3412

**Published:** 2025-12-31

**Authors:** Richard Marottoli, Margaret Doyle, Margaret Wallhagen

**Affiliations:** Yale University School of Medicine, New Haven, Connecticut, United States; Yale University School of Medicine, New Haven, Connecticut, United States; University of California, San Francisco, San Francisco, California, United States

## Abstract

The ability to continue to drive is often essential to an older adult’s independence yet may pose a safety risk. Visual and cognitive impairment have been associated with driving performance and crashes, yet less is known about the potential effects of hearing loss. We examined the association between hearing and on-road driving performance scores among 494 community living adults aged >70. Eligibility criteria included cognitive status of > 18 on the Mini-Mental State Examination and visual distance acuity > 20/70. Hearing was assessed by the whisper test (6 numbers/ear, possible range 0-12 correct). Driving performance was rated on a previously validated 35-item scale derived from the Connecticut Department of Motor Vehicles exam (scored 0-70, higher score better). Participants had a mean age of 78.6; 87% were male. In negative binomial regression models the number of errors on the hearing test was significantly associated with poorer driving scores (Rate Ratio (CI)=1.03 (1.02, 1.05)). This association was attenuated in a model adjusted for potential demographic and clinical confounders, including cognitive function. With the addition of an item indicating whether the driver understood oral instruction, per the evaluator, the adjusted association became only marginally significant. Findings suggest that hearing deficits may be associated with poorer driving performance and possibly mediated by the inability to adequately hear test instructions. Further research is needed to explore this relationship to assure older adults are not disadvantaged during driving tests.

